# Hydrogen in Patients With Corticosteroid-Refractory/Dependent Chronic Graft-Versus-Host-Disease: A Single-Arm, Multicenter, Open-Label, Phase 2 Trial

**DOI:** 10.3389/fimmu.2020.598359

**Published:** 2020-11-25

**Authors:** Liren Qian, Miao Liu, Jianliang Shen, Jian Cen, Defeng Zhao

**Affiliations:** ^1^ Department of Hematology, The Sixth Medical Center, Chinese PLA General Hospital, Beijing, China; ^2^ Department of Statistics and Epidemiology, Graduate School of Medical School of Chinese PLA General Hospital, Beijing, China; ^3^ Department of Hematology, Boren Hospital, Beijing, China

**Keywords:** corticosteroid, hydrogen, graft-versus-host-disease, refractory, chronic graft-versus-host-disease

## Abstract

Chronic graft-versus-host-disease (cGVHD) is the leading cause of late non-relapse mortality after allogeneic hematopoietic stem cell transplantation(HSCT). There is no standard therapy for patients refractory or dependent to corticosteroid treatment. We hypothesized that hydrogen may exert therapeutic effects on cGVHD patients with few side effects. A prospective open-label phase 2 study of hydrogen was conducted. Patients received hydrogen-rich water 4ml/kg orally three times a day. Responses were graded in the skin, mouth, Gastrointestinal(GI), liver, eyes, lungs and joints and fascia every 3 months. A total of 24 patients (median age 27) were enrolled. Of the 24 patients, 18 (75%; 95% CI, 55.1% to 88%) had an objective response. No significant toxicity was observed. The estimated 4-year overall survival rate was 74.7%(95% CI, 54.9%–94.5%). The survival time was significantly prolonged in the response group. The survival rate at 4 years in the response group is significantly higher than the nonresponse group (86.6% vs 0%; p= 0.000132). Hydrogen showed great efficacy on cGVHD patients and long-term administration of hydrogen was not associated with significant toxic effects. The trial was registered at www.ClinicalTrials.Gov, NCT02918188.

## Introduction

Allogeneic hematopoietic stem cell transplantation (allo-HSCT) has been widely used in the treatment of benign and malignant hematopoietic diseases. Chronic graft-versus-host disease (cGVHD) is a very common complication of allo-HSCT and has become the main cause of non-transplant-related death after transplantation (1). Among patients who received allo-HSCT, the cumulative incidence of cGVHD requiring treatment within 2 years after transplantation reaches 30%–40%. With the increased use of allo-HSCT in elderly patients, the widespread use of peripheral blood stem cells as sources and the improvement of the initial survival rate after transplantation, the incidence of cGVHD is increasing year by year (2). However, in the past three decades, the first-line treatment of cGVHD still mainly depends on corticosteroids (such as prednisone, methylprednisolone, dexamethasone, etc.). Clinical initial treatment of cGVHD often uses prednisone with or without cyclosporine or tacrolimus. The initial dose of prednisone is usually 0.5–1 mg/kg/day, which will be gradually reduced after the disease is controlled (3, 4). In the process of glucocorticoid reduction, the symptoms of patients with cGVHD are often aggravated or even relapsed, which leads to the cessation of glucocorticoid reduction or even increase. The duration of treatment of cGVHD with glucocorticoids often reaches 3 months or even longer (5). However, long-term use of glucocorticoids can lead to serious toxic side effects. In order to minimize the toxic side effects of long-term use of glucocorticoids, clinicians often use glucocorticoids in combination with other immunosuppressive agents to treat cGVHD (6), mainly including calmodulin inhibitors (such as cyclosporine, tacrolimus), azathioprine, mycophenolate mofetil (MMF), hydroxychloroquine, etc. However, a number of clinical trials have confirmed that combining azathioprine (7), mycophenolate mofetil (5), hydroxychloroquine (8), thalidomide, etc (9) on the basis of initial treatment did not bring benefits. Fifty percent to 60% of cGVHD patients treated with first-line treatment still need to receive second-line treatment (10). At present, a number of clinical trials on the second-line treatment of cGVHD has been carried out internationally, but the results are often not convincing due to flaws in trial design (6). Clearly, there is an urgent need to find a better treatment for cGVHD (11). In 2007, Ohsawa et al. systematically reported the biological effects of molecular hydrogen (H_2_) for the first time (12). Their study found that H_2_ has a good antioxidant effect. Compared with tacrolimus, H_2_ has the same therapeutic effect on cerebral ischemia-reperfusion injury in rats. The researchers also found that, similar to tacrolimus, H_2_ has a strong anti-inflammatory effect in addition to its antioxidant effect. Tacrolimus has already become a first-line treatment for rejection of solid organ transplantation and cGVHD after allo-HSCT. In addition, H_2_ has also been proven to have a good anti-fibrotic effect. Terasaki Y et al. found in the mouse models of pulmonary fibrosis post radiation that breathing air containing 4% H_2_ has a significant therapeutic effect on chronic pulmonary fibrosis, and can significantly delay the progress of pulmonary fibrosis (13). Although the current mechanism of cGVHD is not very clear, in the currently recognized pathogenesis of cGVHD, inflammatory factors imbalance and fibrosis occupy a dominant position (4, 14). Since H_2_ has anti-inflammatory and anti-fibrosis effects, we speculate that H_2_ may have potential therapeutic effects for cGVHD after allo-HSCT. We have proved the its therapeutic effects in a mouse model preliminary (15). To further verify the hypothesis, we conducted a multiple center study of hydrogen in patients with corticosteroid-refractory/dependent cGVHD to look at response rate and to detect any unique toxicities.

## Subject and Methods

### Patient Eligibility

This investigator-initiated study was approved by the ethics committee of the Sixth Medical Center, Chinese People’s Liberation Army (PLA) General Hospital, China. All of the patients or their legal guardians signed informed consent forms in accordance with the Declaration of Helsinki. This trial has been registered as NCT02918188 (www.clinicaltrials.gov).

Patients who underwent allogeneic stem cell transplantation were enrolled. Eligible patients met the following criteria: patients younger than 65 years diagnosis of steroid-refractory cGVHD (no response after prednisone at least 1 mg/kg or equivalent dose of another steroid or another immunosuppressive regimen) or steroid-dependent cGVHD (had an initial response followed by a cGVHD flare upon steroid taper). A tissue biopsy before entering the study with histology consistent with cGVHD was required unless there was a medical contraindication such as concern for poor wound healing after the biopsy. Exclusion criteria were: patients with other stable diseases(not chronic GVHD), not well controlled by the current treatment; pregnancy; HIV positive; severe liver or renal impairment: serum creatinine >2.5 mg/dl, serum bilirubin>2.5 mg/dl (without evidence of hepatic cGVHD); uncontrolled malignancies including the persistence of the underlying malignancy before the allogeneic transplantation and the relapse of hematopoietic malignancy; any other investigational agents administered within last four weeks; cardiac insufficiency (>grade II, New York Heart Association classification); inability to comply with medical therapy or follow-up.

### Therapy

Patients included in this study received hydrogen-rich water as their only new intervention. Hydrogen-rich water (0.8 ppm H_2_) was administered orally three times a day at 4 ml/kg. The hydrogen-rich water (0.8 ppm H_2_) was supplied by Beijing Huoliqingyuan Beverage Co., Ltd. (Beijing, China) as previously described (16). All patients received 12 months of therapy unless they required earlier removal from the study. It was recommended that a corticosteroid taper be started between 8 and 12 weeks after initiating hydrogen-rich water. A reduction of 25% of the initial dose every 2 weeks was the recommended corticosteroid taper. It was also recommended that if subjects were receiving a calcineurin inhibitor at the time of study initiation that they remain on it through the duration of the study unless there was drug toxicity. All other immunosuppressants were to be tapered on an individual basis.

### Evaluation of Response and Toxicity

Subjects were evaluated at baseline and then every 3 months using the form according to National Institutes of Health (NIH) Consensus for cGVHD (17). In each subjects, seven domains were assessed: mouth, gastrointestinal, lungs, liver, joint, skin, and ocular. Each domain’s therapeutic response was measured according to the NIH consensus on response criteria (17). No response was defined as disease progression, no change, mixed response (response in 1 or more domains, progress in 1 or more domains) or initial response followed by disease progression. Therapeutic response for each subject was followed 12 months until the completion of the study. Toxicity was assessed every 3 months using Common Toxicity Criteria Version 3.0 according to the National Cancer Institute Common Toxicity Criteria.

### Removal From Protocol

Patients were removed from the study if cGVHD progressed after 12 or more weeks of treatment. Patients were also removed if they were lost to follow-up or if the family withdrew consent. For subjects that were removed from the study, the last assessment before removal was used to determine response. Subjects that were removed are counted as nonresponders. Two patients were removed from this study both due to loss of follow-up.

### Statistics

The primary statistical endpoint was overall response rate (defined as complete or partial response). Subjects were evaluated at baseline and then every 3 months using the form according to National Institutes of Health (NIH) Consensus for cGVHD (17). Overall response was measured according to the NIH consensus on response criteria (17). No response was defined as disease progression, no change, mixed response (response in 1 or more domains, progress in 1 or more domains) or initial response followed by disease progression. A sample size of 17 was calculated according to have 80% power to detect a response rate of at least 50% (11), compared with a control response rate of 20%, with 80% power and a type I error rate of 5%. Although this design required 17 patients, the final sample size was 24.

A secondary outcome was response rate in each domain measured in subjects that had had initial involvement in that domain. In each subjects, seven domains were assessed: mouth, gastrointestinal, lungs, liver, joint, skin, and ocular. Each domain’s therapeutic response was measured according to the NIH consensus on response criteria (17). Response rates were calculated together with 95% exact binomial confidence intervals. Descriptive characteristics are shown with percentages or with medians and ranges, as appropriate. Logistic Regression analysis was used to determine significance of certain factors such as history of acute GVHD and severity stage of chronic GVHD on final response. Time to initial response was analyzed using cumulative incidence (18). Survival percentages and confidence intervals were calculated using Kaplan-Meier curves. Curves were compared using the log-rank test. All analyses were performed with SAS (SAS Institute) and SPSS.

## Results

### Patient Characteristics

Twenty-four patients from four different institutions were enrolled. Two patients were removed from the study because of loss of follow-up. Patient characteristics at the entry were given in **Table 1**. Median age of the patients was 27 years old. Patients were diagnosed as acute myeloid leukemia/myelodysplasia (n=12), acute lymphoblastic leukemia(n=10), systemic lupus erythematosus (n=1) and hemophagocytic lymphohistiocytosis (n=1). All patients received myeloablative hematopoietic stem cell transplantation. Subjects had received different donor stem cell sources: haploidentical bone marrow plus peripheral blood stem cell (PBSC) (n=13), human leukocyte antigen (HLA)-identical sibling bone marrow plus PBSC (n=5), HLA-identical sibling PBSC (n=3), unrelated donor PBSC (n=1), unrelated cord blood (n=1), and haploidentical PBSC (n=1).

**Table 1:** Patient characteristics at study entry (N=24).

	Values	Responders	Non-responders
Median Age, y(range)*	27(9–54)	27(9–54)	32.5(21–37)
Gender*			
Male, n (%)	12(50.0)	10(55.6)	2(33.3)
Female, n (%)	12(50.0)	8(44.4)	4(66.7)
Diagnoses*			
Acute myeloid leukemia/myelodysplasia, n (%)	12 (50.0)	9(50.0)	3(50.0)
Acute lymphoblastic leukemia, n (%)	10 (41.7)	7(38.9)	3(50.0)
Systemic lupus erythematosus, n (%)	1 (4.2)	1(5.6)	0
Hemophagocytic lymphohistiocytosis, n (%)	1 (4.2)	1(5.6)	0
Transplantation regimen*			
Myeloablative, n (%)	24 (100)	18(100)	6 (100)
Stem cell Source*			
Haploidentical bone marrow + PBSC, n (%)	13 (54.2)	10(55.6)	3(50.0)
HLA-identical sibling bone marrow + PBSC, n(%)	5 (20.8)	3(16.7)	2(33.3)
HLA-identical sibling PBSC, n(%)	3 (12.5)	2(11.1)	1(16.7)
Unrelated donor PBSC, n(%)	1 (4.2)	1(5.6)	0
Unrelated cord blood, n(%)	1 (4.2)	1(5.6)	0
Haploidentical PBSC, n(%)	1 (4.2)	1(5.6)	0

HLA, human leukocyte antigen; PBSC, peripheral blood stem cell. The responders compared with non-responders *P > 0.05.

The chronic GVHD characteristics of the subjects at study entry are given in **Table 2**. Median beginning platelet count was 168, and 33.3% of the subjects are with platelet count less than 100×10^9^/L. 79.2% of the subjects at study entry are on corticosteroids and median beginning prednisone dose was 0.21 mg/kg/day. 66.7% of the subjects had a history of acute GVHD. 79.2% had severe chronic GVHD according to the NIH global severity stage (17). The Karnofsky performance scale (KPS) of 10 subjects is no greater than 80.

**Table 2:** Chronic GVHD characteristics at study entry.

	Values	Responders	Non-responders
Median beginning platelet count, 10^9^/L (range) *	168(31–428)	182.5(31–428)	142.5 (33–175)
Median beginning prednisone dose, mg/kg/d (range) *	0.21(0–0.81)	0.185(0–0.81)	0.27(0–0.48)
Subjects on corticosteroids at study entry, n (%)*	19(79.2)	14(77.8)	5(83.3)
Subjects with platelet count less than 100×10^9^/L, n (%)*	8(33.3)	6(33.3)	2(33.3)
Performance score no greater than 80, n (%)	10(41.7)	7(38.9)	3(50.0)
History of acute GVHD, n (%)*	16 (66.7)	11(61.1)	5(83.3)
Severe chronic GVHD (NIH global severity stage), n (%)*	19 (79.2)	14(77.8)	5(83.3)

GVHD, graft-versus-host disease; NIH, National Institutes of Health. The responders compared with non-responders *P > 0.05.

### Response

At the entry of the study, skin was the most involved (75.0%), followed by the gastrointestinal tract (70.8%), while liver is the least involved organ(8.3%). 66.7% of patients had clinical manifestations of cGVHD in mouth, 63% of patients manifestated in the eyes, 33.3% in lungs, and 29.2% in joints and fascia. Overall, of the 24 patients, 18 (75.0%; 95% CI, 55.1% to 88%) had an objective response to hydrogen therapy at the last evaluation. Eight patients had complete response, 10 had partial response, five had disease progression, and one had stable disease. Of the 18 patients who responded to treatment, 15 had a response at 30 days of treatment, two at 90 days, one at 180 days. All these 18 patients had sustained response at last evaluation. The median time to response was 30 days (range, 30 to 180 days).

Of the 24 patients enrolled, 18 patients received therapy for 12 months. Two patients were removed from the study both due to loss of follow-up. Four patients died within 12 months. Of the 18 patients who received therapy for 12 months, eight had complete response, nine had partial response, and one had disease progression. The percentage of patients who received 12 months of therapy and had a response was 17/24 (70.8%; 95% CI, 50.8% to 85.1%).

Among the 16 patients with cGVHD affecting the oral cavity, 10 patients had complete remission and three patients had partial remission. The response rate was 81.25% (95% CI, 57.0%–93.4%). Of the 17 patients with cGVHD affecting gastrointestinal tract, 13 had complete remission and one had partial remission. The response rate was 82.35% (95% CI, 59.0%–93.8%). The detailed response rates in the various organs are listed in **Table 3**. Among them, the response rate of lung is the lowest, 50% (95% CI, 21.5%–78.5%), and the response rate of liver is the highest, 100% (34.2%-100%). Detailed grades of the cGVHD per organ at study entry and one year after or last assessment for each patient is showed in **Table 4**. We also used the Karnofsky performance scale (KPS) as an indicator of the patient’s quality of life. Our study found that among patients who continued to drink hydrogen-rich water for more than 12 months, KPS has been improved (p=0.034).

**Table 3:** Response by domain (N=24).

	Involved, n (%)	Complete Response, n (%)	Partial Response, n (%)	Stable, n (%)	Progression, n (%)	Response rate, % (95% CI)*
**Mouth**	16 (66.7)	10(62.5)	3(18.8)	1(6.3)	2(12.5)	81.25(57.0–93.4)
**Gastrointestinal**	17 (70.8)	13(76.5)	1(5.9)	0(0)	3(17.6)	82.4(59.0–93.8)
**Lung**	8 (33.3)	4(50.0)	0(0)	2(25.0)	2(25.0)	50.0(21.5–78.5)
**Liver**	2 (8.3)	1(50)	1(50)	0(0)	0(0)	100(34.2–100)
**Skin**	18 (75.0)	11(61.1)	1(5.6)	2(11.1)	4(22.2)	66.7(43.8–83.7)
**Ocular**	17 (63.0)	9(52.9)	2(11.8)	3(17.6)	3(17.6)	64.7(41.3–82.7)
**Joints and fascia**	7 (29.2)	5(71.4)	0(0)	1(14.3)	1(14.3)	71.4(35.9–91.8)

CI, confidence interval. *Response rate included CR + PR.

**Table 4:** Severity score of chronic graft-versus-host-disease (cGVHD) per organ at study entry and one year after or last assessment.

Patient number	Mouth at the entry	Mouth at one year or last assessment	Gastrointestinal at the entry	Gastrointestinal at one year or last assessment	Lung at the entry	Lung at one year or last assessment	Liver at the entry	Liver at one year or last assessment	Skin at the entry	Skin at one year or last assessment	Ocular at the entry	Ocular at one year or last assessment	Joints and fascia at the entry	Joints and fascia at one year or last assessment
1	3	2	2	0	0	0	0	0	2	0	1	0	0	0
2	0	0	0	0	0	0	0	0	3	0	0	0	0	0
3	0	0	0	0	2	2	0	0	0	0	2	0	0	0
4	2	2	3	0	2	0	0	0	0	0	0	0	1	0
5	3	2	2	0	0	0	0	0	0	0	1	0	2	0
6	2	0	1	0	2	0	0	0	3	0	3	3	0	0
7	1	0	1	0	0	0	0	0	2	0	1	0	0	0
8	1	0	1	0	0	0	0	0	3	0	3	0	0	0
9	1	0	1	2	0	0	0	0	2	1	0	0	1	1
10	3	2	1	0	0	0	0	0	0	2	2	2	0	0
11	3	0	3	0	0	3	0	0	3	0	1	0	0	0
12	2	0	0	0	0	0	0	0	3	0	1	0	0	0
13	0	0	1	0	0	0	0	0	1	0	0	0	0	0
14	3	0	2	0	1	0	0	0	3	0	3	2	1	0
15	3	0	1	0	0	0	0	0	0	0	1	0	0	0
16	0	0	1	0	0	0	0	0	2	0	0	0	1	0
17	0	0	3	2	0	0	3	0	0	0	0	0	0	0
18	2	0	0	0	2	3	0	0	1	1	2	2	0	0
19	0	0	0	0	2	0	2	1	0	0	0	0	0	0
20	2	0	0	0	0	0	0	0	3	3	3	1	0	0
21	0	0	1	0	1	1	0	0	1	0	2	0	1	0
22	3	3	2	3	0	0	0	0	3	3	3	3	1	3
23	2	3	1	3	0	0	0	0	3	3	1	3	0	0
24	0	0	0	0	0	0	0	0	3	3	3	3	0	0

**Figure 1:**
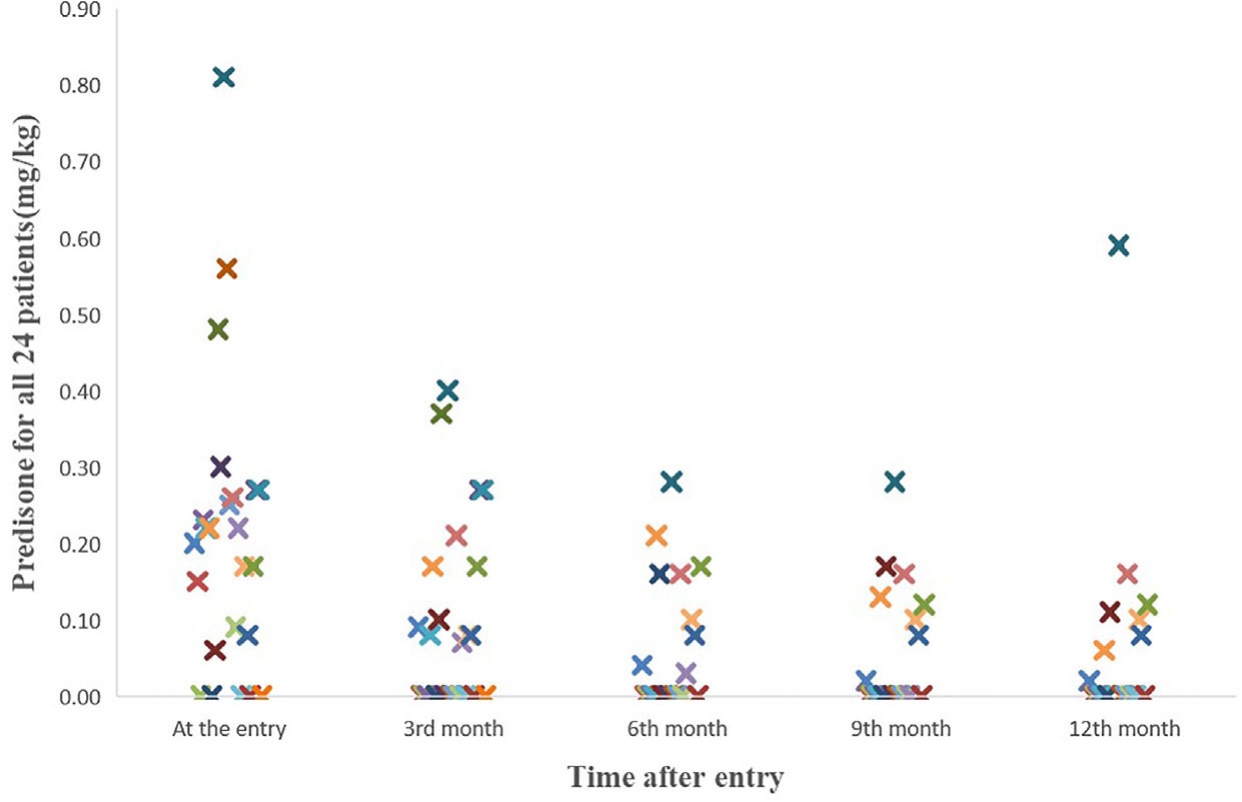
The course of the prednisone doses for all 24 patients. The median beginning prednisone dose in the response group was 0.185 mg/kg/d (0-0.81mg/kg/d) while the median end prednisone dose was 0 mg/kg/d (0-0.59mg/kg/d; P=0.004).

We also evaluated whether hydrogen therapy had a corticosteroid sparing effect in patients who responded. The median beginning prednisone dose in the response group was 0.185 mg/kg/day (0–0.81 mg/kg/day) while the median end prednisone dose was 0 mg/kg/day (0–0.59 mg/kg/day; P=0.004) (**Figure 1**). In the six patients who did not respond to hydrogen therapy, the median beginning prednisone dose in the non-response group was 0.27mg/kg/day (0–0.48 mg/kg/day) while the median end prednisone dose was 0.05 mg/kg/day (0–0.27 mg/kg/day). The change in non-response group was not statistically significant (P =0.115). Besides, as shown in **Table 5**, there were 13 patients taking immunosuppressive drugs at the entry and one year after study entry among the 18 patients who reached this time point.

**Table 5:** Immunosuppressive drugs taken at the entry and one year after study entry for subjects reached this time point.

Patients	At entry	One year after study entry
1	Prednisone	Prednisone
2	Prednisone + CSA	FK506
3	None	None
4	Prednisone + CSA	MMF
5	Prednisone + CSA	CSA
6	Prednisone + FK506	Prednisone + FK506
7	FK506	FK506
8	Prednisone + CSA	Prednisone
9	Prednisone + FK506	\
10	Prednisone + FK506	\
11	Prednisone + CSA	Prednisone + FK506
12	Prednisone + FK506	None
13	Prednisone + FK506	MMF + FK506
14	Prednisone + FK506	Prednisone
15	Prednisone + CSA	None
16	Prednisone	None
17	None	\
18	Prednisone + FK506	Prednisone + FK506
19	Prednisone + FK506	Prednisone + FK506
20	Azathioprine + MTX + MMF	None
21	Prednisone + FK506	Prednisone + FK506
22	Prednisone + CSA + MMF	\
23	Prednisone + CSA + MMF	\
24	None	\

FK506, Tacrolimus; MMF, Mycophenolate Mofetil; CSA, Cyclosporine A

### Toxicity and Mortality

**Figure 2:**
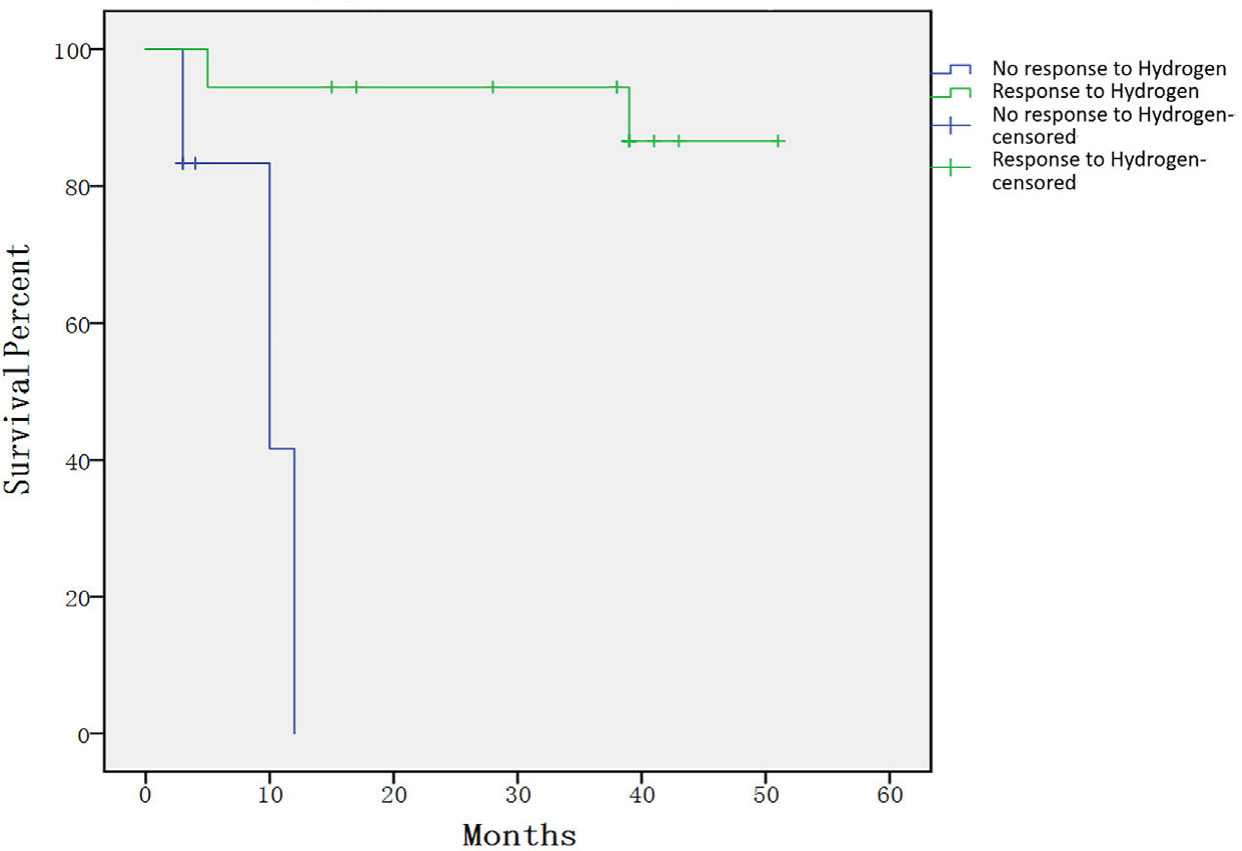
Individual survival curves shown for subjects that had response compared with those that did not. The 4-year projected survival rate was 86.6% (95% CI, 69.0%–100%) among patients who responded to treatment, which was significantly higher than that in the group did not respond to treatment (p= 0.000132).

None of the enrolled patients were removed from the study due to toxicity. No obvious toxicity and side effects were seen in 24 enrolled patients. Only three patients had transient hiccups. Three patients died during treatment, one case was due to a serious fungal infection. One-year survival rate of the entire cohort was 81.5% (95% CI, 65.0%–98.0%). The 4-year projected survival rate of the entire cohort was 74.7% (95% CI, 54.9%–94.5%).The 4-year projected survival rate was 86.6%(95% CI, 69.0%–100%) among patients who responded to treatment, which was significantly higher than that in the group did not respond to treatment(p= 0.000132), as shown in **Figure 2**. The median follow-up time was 38.5 months (range, 3–51 months).

## Discussion

In this study, we evaluated the response rate and toxicity of molecular hydrogen on patients with chronic GVHD who are corticosteroid-refractory/dependent. The study found a greater than 50% response rate, which reached its primary endpoint.

Results of the study are encouraging, mainly due to the following points: Firstly. It has been confirmed that molecular hydrogen has a good response rate in chronic graft-versus-host patients, and the overall response rate can reach 74.7%. However, the response rate reported in the previous literature fluctuated between 40% and 60% (11, 19). Secondly, Hydrogen is not toxic. Treating chronic GVHD with immunosuppressive agents can increase the risk of infection. However, in polymicrobial sepsis models, molecular hydrogen has been proved to have anti-infective effects but not increasing the risk of infection (20–22), which is different from immunosuppressive agents. In our study, only three patients experienced transient hiccups and no other side effects. Hydrogen and helium mixed gas has been used in diving for a long time, and divers have no obvious adverse reactions even when breathing high-pressure hydrogen (23). Besides, bacteria in the colon of humans and animals can also produce a certain level of H_2_ (12). These all indicate that H_2_ has no toxic side effects on the human body. Moreover, H_2_ in the body can be discharged through the respiratory system without any residue. This characteristic determines that H_2_ can be used for a long time, and this characteristic can precisely target the long-term, repeated and protracted disease characteristics of cGVHD. It was found in the study that the median response time is 30 days, and the response rate gradually increases with the extension of the treatment time. The study was designed to administer hydrogen continuously for 1 year. We believe that if the administration time is extended, the overall response rate will still rise. Thirdly, the hydrogen molecule is very small, has a strong penetration ability, can quickly penetrate the biofilm, and reach a higher concentration in the cell to play a therapeutic role (24). Finally, H_2_ is cheap and easy to obtain. H_2_ is widely distributed in nature, and the industrial preparation technology is very mature and the price is very low. A considerable part of the existing cGVHD drugs (such as tacrolimus, Ixazomib (25), ibutinib (26, 27), ruxolitinib (28), etc.) are very expensive, making many families poorer due to illness.

In our study, we can see that the response rate of the digestive system is higher, the response rate of the oral cavity can reach 81.25%, and the response rate of the gastrointestinal tract can reach 82.4%. Both patients with liver involvement have obtained a response. We considered that the overall repoonse rate is higher because the hydrogen-rich water can directly act on the digestive system due to the higher hydrogen concentration in the digestive system. Therefore, we speculate that patients with lung involvement can use the method of breathing hydrogen, patients with eye involvement can use hydrogen-rich eye drops, and patients with skin involvement can use hydrogen-rich water to wash, which may achieve better treatment results.

Although the long-term outcome is not a primary endpoint of the study, our research indicates that the 4-year projected overall survival rate of 74.7% is encouraging. Recently, the number of studies on chronic GVHD have gradually increased (19, 25, 29, 30), but few publications report the long-term survival rate of corticosteroid-refractory/dependent cGVHD. In a newly diagnosed children study, the 3-year survival rate is approximately 70% (31). In a corticosteroid-refractory cGVHD study, the 3-year survival rate is approximately 60% (11). Therefore, in our study, the four-year survival rate of 74.7% is very exciting. Compared with the group that did not respond to H_2_, the survival rate of patients who responded to hydrogen treatment was significantly improved. Therefore, we can use the response to hydrogen therapy as a potential prognostic indicator. In our study, all patients in the group that did not respond to hydrogen therapy died within 1 year, which may be due to the seriousness of the conditions of the enrolled patients, because patients with severe cGVHD accounted for nearly 80% at entry of study.

In conclusion, this study for the first time suggested effectiveness of hydrogen therapy in patients with corticosteroid-refractory/dependent cGVHD compared to historical controls. The overall response rate can reach 75%, with almost no toxic side effects, which can precisely target the disease characteristics of cGVHD. Its long-term survival rate is encouraging. We strongly support the future multi-institutional research in the world. Whether molecular hydrogen can be used as the first-line treatment of cGVHD still requires further clinical trials.

## Data Availability Statement

The original contributions presented in the study are included in the article/supplementary material. Further inquiries can be directed to the corresponding author.

## Ethics Statement

The studies involving human participants were reviewed and approved by the ethics committee of the Sixth Medical Center, Chinese PLA General Hospital, China. Written informed consent to participate in this study was provided by the participants’ legal guardian/next of kin.

## Author Contributions

LQ and ML designed the experiment, analyzed data, and wrote the paper. JS, JC, and DZ performed the experiments and analyzed data. All authors contributed to the article and approved the submitted version.

## Funding

This work was supported by a grant from the National Natural Science Foundation of China (Grant No. 81800180).

## Conflict of Interest

The authors declare that the research was conducted in the absence of any commercial or financial relationships that could be construed as a potential conflict of interest.
